# The crucial role of density functional nonlocality and on-axis CH_3_NH_3_ rotation induced I_2_ formation in hybrid organic-inorganic CH_3_NH_3_PbI_3_ cubic perovskite

**DOI:** 10.1038/s41598-018-31462-x

**Published:** 2018-09-03

**Authors:** Rakchat Klinkla, Vichawan Sakulsupich, Teerachote Pakornchote, Udomsilp Pinsook, Thiti Bovornratanaraks

**Affiliations:** 1 Extreme Conditions Physics Research Laboratory, Physics of Energy Materials Research Unit, Department of Physics, Faculty of Science, Chulalongkorn University, Bangkok, 10330 Thailand; 2 Thailand Center of Excellence in Physics, Commission on Higher Education, 328 SiAyutthaya Road, Bangkok, 10400 Thailand

## Abstract

Effects of electronic nonlocality in density functional theory study of structural and energetic properties of a pseudocubic CH_3_NH_3_PbI_3_ are investigated by considering coherent rotation around C–N axis of a CH_3_NH_3_ cation. A number of truly non-local and semi-local exchange correlation density functionals are examined by comparing calculated structural parameters with experimental results. The vdW-DF-cx which takes into account the non-local van der Waals correlation and consistent exchange shows the best overall performance for density functional theory study of this system. Remarkable distinctions between results from vdW-DF-cx and those from PBEsol exchange correlation functionals are observed and indicate the need of including the non-local interaction in the study of this system, especially its dynamical properties. The obtained rotational barriers are 18.56 meV/formula and 27.71 meV/formula which correspond to rotational frequencies of 3.71 THz and 2.60 THz for vdW-DF-cx and PBEsol calculations, respectively. Interestingly, the maximally localised Wannier function analysis shows the hydrogen bonding assisted covalent character of two iodide anions at a moderate rotational angle which can lead to I_2_ formation and then material degradation.

## Introduction

In the last decade, there has been a booming interest in hybrid organic inorganic perovskites (HOIPs) originates from their potential applications in optoelectronic, thermoelectric, and photovoltaic technologies, owing to their favourable electronic, excitonic and optical properties^1–4^. In only ten years’ time, reported power conversion efficiencies of perovskite solar cells have been raised up from the first record of 2.2% in 2009, to 22.1% in 2016^2,5,6^. Intensive researches have rendered perovskite solar cells into devices that are both easy to fabricate and have high cell power conversion efficiency, on par with those of silicon solar cells^7^. But commercial perovskite photovoltaic devices are still several years down the road, due to its stability issues^8–11^.

HOIPs are in a class of material that has a perovskite crystal structure with an ABX_3_ chemical composition, where A, B, and X represent an organic monovalent cation, an inorganic cation and a halogen, respectively. Methylammonium lead iodide (MAPbI_3_) perovskite, which, as the name suggested, has methylammonium (MA) as the cation, is regarded as the archetype of HOIPs since it exhibits rich fundamental photovoltaic properties, and has recently been intensively studied and developed for solar cell devices. MAPbI_3_ exists in three phases, namely orthorhombic (Pnma) at 0 K < *T* < 165 K, tetragonal (I4/mcm) at 165 K < *T* < 327 K, and cubic (Pm $\bar{3}\,$m) at *T* > 327 K with ordered antiferroelectric MA cation arrangement for the first, and disordered arrangements for the latter two^12–15^. Orientation dynamics of the organic cations are associate with physical properties of MAPbI_3_. One is the ferroelectric effect which is related to an anomalous hysteresis in the HOIP solar cells^16^. Long migration length of electrons and holes^17^, structural evolution and stability also result from the said dynamics^14,15,18–24^.

Recent experimental results suggest that there exist two kinds of dynamics associated with a MA cation in MAPbI_3_^23^. The first type is the reorientation of the C–N axis with estimated 3–14 ps time scale^16,17,19,23^. The second type is the much faster methyl and/or ammonium groups rotation about C-N axis with sub-picosecond time scale^25^ that has recently been estimated at ~30 times faster^26^. Such fast-rotation cannot be precisely probed by experiments^23^. Moreover, the interplay between octahedron tilting and the MA dynamics still needs a clearer understanding, as it has recently been dubbed as the chicken-and-egg paradox by Li and Rinke^27^.

Since the emergence of HOIPs as photovoltaic materials in 2009^2^, density functional theory (DFT) based calculations have been adopted to study their properties and underlying physics. Most of the studies are based on the standard semi-local generalised gradient approximation which cannot retain the non-local nature of electron correlation. However, many suggest the importance of including the non-local van der Waals (vdW) interaction in the calculations to recover such many-body effect^24,27–31^. Nevertheless, there are a number of methods for including the vdW-interaction implemented in the DFT based software packages. Each of them yields different levels of accuracy, especially, for molecular, sparse and hydrogen-bond materials. The detailed discussions on the methodology and accuracy of the vdW density functionals (vdW-DFs) can be found elsewhere^32–34^.

In this work, we examine the application of various vdW-DFs and discuss in detail the equilibrium structural configurations corresponding to the on-axis MA rotation obtained by structural relaxations. As a result, vdW-DF-cx non-local exchange correlation (xc) functional shows the best overall performance for this system with some remarkable differences in the atomic configurations comparing with those of PBEsol semi-local xc-functional. By adding the non-local effect, lowering and broadening of the rotational energy barrier are observed. Furthermore, maximally localised Wannier function (MLWF) analysis illustrates the formation of hydrogen bond assisted I-I covalent bonding at a specific MA rotational angle which could lead to the formation of neutral I_2_ defect and so the material degradation.

## Results and Discussion

### Structural parameters

First, we examine the xc-functionals by calculating structural parameters of the pseudocubic MAPbI_3_ unit cell. Since the assigned Pm $\bar{3}\,$m symmetry of MAPbI_3_ was derived from the average structure of the material at high temperature. Therefore, the average values of such calculated parameters, i.e. average lattice constant ($\bar{{\rm{a}}}$), average Pb-I bond length over three inequivalent I-atoms ($\overline{{\rm{PbI}}}$), average C-H bond length ($\overline{{\rm{CH}}}$), average N-H bond length ($\overline{{\rm{NH}}}$), and C-N bond length (CN) obtained by performing full structural relaxation, there is no any constraint, where the C-N axis is at the lowest enthalpy arrangement^14^, are used for the comparison with Weller *et*. *al*.’s accurate powder neutron diffraction experimental results^15^.

The structural parameters are grouped into two subgroups according to magnitudes of their absolute errors, *i*.*e*. $\bar{{\rm{a}}}$ and $\overline{{\rm{PbI}}}$ as one subgroup and CN, $\overline{{\rm{CH}}}$ and $\overline{{\rm{NH}}}$ as the other. The mean absolute error (MAE) of each subgroup is given in Table 1. The parameters $\bar{{\rm{a}}}$ and $\overline{{\rm{PbI}}}$ are in excellent agreement with experimental results (MAE(1) < 1%) for most of xc-functionals except for vdW-DF, vdW-DF2, and PBE. Notably, the vdW-DF-cx shows the best performance in predicting the lattice constant and the Pb-I distance, see MAE(1) in Table 1. This is in good agreement with the vdW-DF testing performed by Berland and Hyldgaard. They examined the accuracy of a number of vdW-DFs in predicting the binding energy and equilibrium separation in H-bond and dispersive-force dominated systems and found that vdW-DF-cx shows the best overall performance^32^. However, MAE(2) in Table 1 shows that errors in CN, $\overline{{\rm{CH}}}$, and $\overline{{\rm{NH}}}$ are in approximately the same order for all xc-functionals, with an error of ~10% for CN and $\overline{{\rm{CH}}}$, and ~5% for $\overline{{\rm{NH}}}$. The disordered C-N axis orientation and the fast on-axis rotation of H-atoms could probably be responsible for the errors in structural parameters. The divergence from the experimental data might originate from the indistinguishability of the atomic position reported by the experiment. Since the position of C, N, H_C_ and H_N_ atoms cannot be experimentally captured due to the MA dynamics, their positions are approximately assigned to Wyckoff sites 4MM(100) and 1 of the Pm $\bar{3}\,$m space group^15^. From the simulation viewpoint, by using only one pseudocubic primitive unit cell, the disordered nature of the organic cations is neglected, giving rise to the divergence from averaged value of the experimental data. Moreover, the effects of temperature which drive atoms from their zero-Kelvin equilibrium configurations are also not included. Although the simulations give large MAE(2), the lattice constant and the Pb-I bond length are in good agreement with those of the experimental results, thanks to their low dynamics. The latter validates the use of computational model for investigating the local structural and energetic responses to the rotation of H atoms about the C-N axis.

**Table 1:** Calculated average structural parameters using semi-local and non-local vdW-DFs compared with experimental results^15^.

Structural parameters (A^°^)	$\bar{{\bf{a}}}$	$\overline{{\bf{P}}{\bf{b}}{\bf{I}}}$	**MAE(1)**	**CN**	$\overline{{\bf{C}}{\bf{H}}}$	$\overline{{\bf{N}}{\bf{H}}}$	**MAE(2)**
PBE	6.471(0.2)	3.205(0.05)	0.1000	1.492(0.1)	1.094(0.1)	1.040(0.05)	0.097
PBEsol	6.299(0.02)	3.146(0.01)	0.0156	1.484(0.1)	1.100(0.1)	1.047(0.05)	0.098
rVV10	6.373(0.06)	3.183(0.02)	0.0404	1.503(0.2)	1.093(0.1)	1.040(0.05)	0.100
vdW-DF	6.554(0.2)	3.237(0.08)	0.1576	1.513(0.2)	1.090(0.1)	1.036(0.04)	0.101
vdW-DF2	6.562(0.3)	3.258(0.1)	0.1721	1.524(0.2)	1.087(0.1)	1.036(0.04)	0.104
vdW-DF2-b86r	6.357(0.04)	3.173(0.01)	0.0268	1.496(0.2)	1.097(0.1)	1.043(0.05)	0.100
vdW-DF-cx	6.320(0.003)	3.149(0.01)	0.0061	1.493(0.2)	1.098(0.1)	1.044(0.05)	0.100
vdW-DF-ob86	6.329(0.01)	3.153(0.006)	0.0083	1.497(0.2)	1.097(0.1)	1.043(0.05)	0.100
Exp.	6.317(0.0)	3.159(0.0)	—	1.348(0.0)	0.994(0.0)	0.994(0.0)	—

The numbers in parentheses stand for deviation of calculated values from experimental ones. MAE(1) and MAE(2) are mean absolute error of subgroups of parameters $\{\bar{{\rm{a}}},\,\overline{{\rm{PbI}}}\}$ and $\{{\rm{CN}},\,\overline{{\rm{CH}}},\,\overline{{\rm{NH}}}\}$, respectively.

### Energy barriers, Equilibrium atomic configurations, and H-bonds

**Figure 1:**
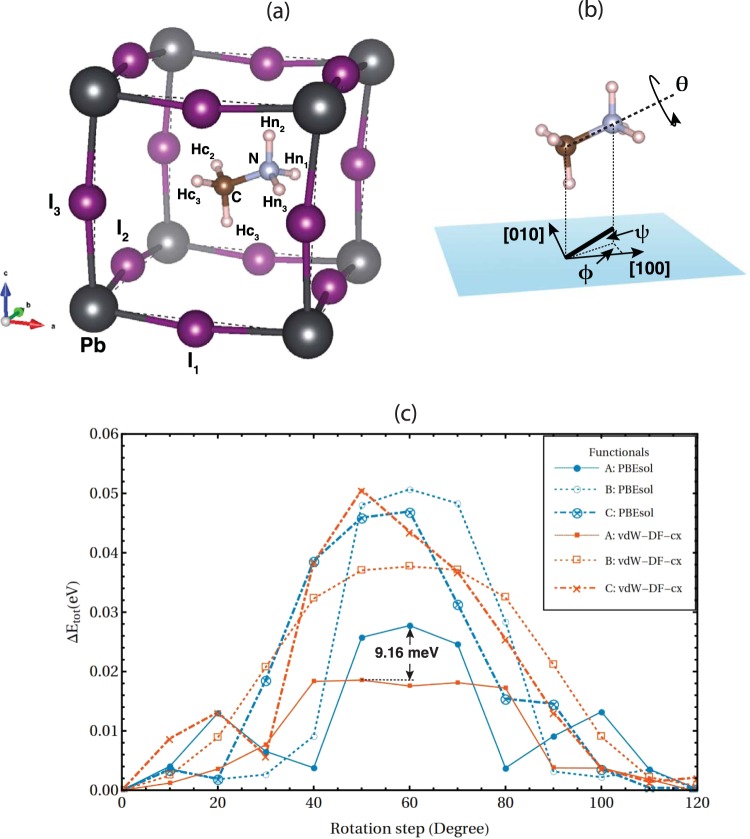
(**a**) Atomistic model of a unit cell. (**b**) Parameters that describe MA orientation ($\varnothing ,\,{\boldsymbol{\psi }}$), and rotation (***θ***). (**c**) Rotational barriers obtained by performing structural relaxation with: (A) no any constraint, (B) fixed cell parameters at initial structure, and (C) fixed cell parameters and I-atomic position at initial structure.

The rotational energy barriers are obtained by performing structural relaxation without any constraint after coherent H-atoms rotation (solid lines in Fig. 1(c)). The rotation of MA is discretised to 12 rotational steps with 10° step size owing to its *C*_3*v*_ symmetry, see Fig. 1 for atomistic structural model. Hereafter, only the results from vdW-DF-cx and PBEsol xc-functionals are shown as they are the representatives of non-local and semi-local xc-functionals respectively, results from all xc-functionals are provided in supplementary information. According to Fig. 1(c), the rotational potential energy, ${\rm{\Delta }}E(\theta )=E(\theta )-E({0}^{o}),$ illustrates that the vdW-DF-cx energy barrier $({\rm{\Delta }}{E}^{{\rm{v}}{\rm{d}}{\rm{W}}-{\rm{D}}{\rm{F}}-{\rm{c}}{\rm{x}}}({50}^{o})\cong 18.56$ meV/formula) is 9.16 meV/formula lower than that of PBEsol (${\rm{\Delta }}{E}^{{\rm{PBEsol}}}({60}^{o})\cong 27.71$ meV/formula) and the vdW-DF-cx rotational potential energy curve is broader than that of PBEsol.

**Figure 2:**
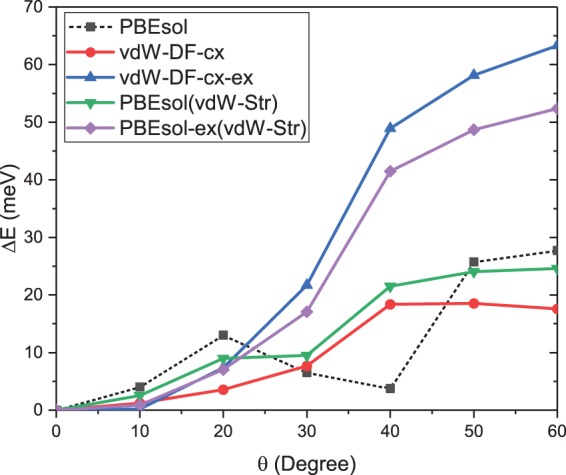
Rotational potential energy curves of the vdW-DF-cx-optimised structure calculated with vdW-DF-cx (red), PBEsol (green), PBEsol with gradient correction correlation off (purple), and vdW-DF-cx with nonlocal correlation off (blue). Dashed line is the rotational potential energy curve of the PBEsol-optimised structure.

**Figure 3:**
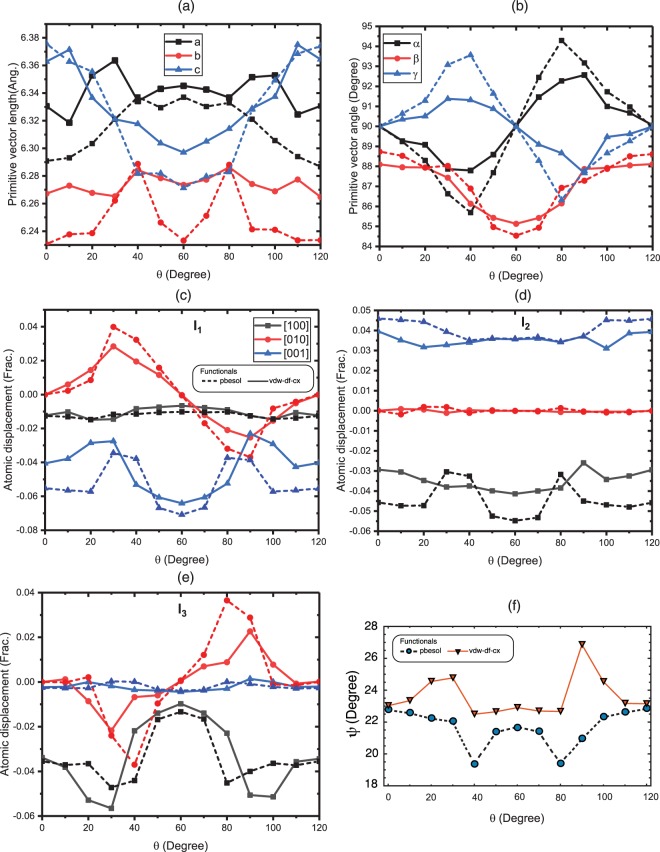
(**a**) Magnitudes of primitive vectors, (**b**) angles between them, and (**c**–**e**) atomic displacements (from Pb – I bond center) in crystallographic directions of three inequivalent I-atoms calculated by applying vdW-DF-cx (solid) and PBEsol (dashed) xc-functionals. (**f**) Altitudinal angle of C–N axis.

**Figure 4:**
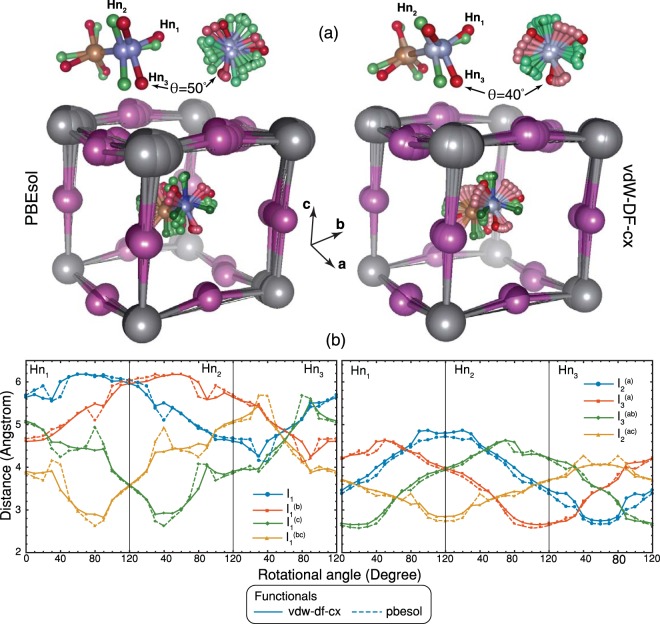
(**a**) Atomistic representations of optimised structures with PBEsol (left), and vdW-DF-cx (right) xc-functionals for ***θ*** = 10° to 80° illustrate the abrupt changes in H-atom positions at *θ* = 50° and *θ* = 40° obtained by applying PBEsol and vdW-DF-cx xc-functionals, respectively. (**b**) H-I distances obtained by structural relaxation with application of vdW-DF-cx (solid) and PBEsol (dashed) xc-functionals where I_i_^(xyz)^ represent a replication of the I_i_ – atom obtained by performing translational symmetry operation with x, y, and z primitive vectors.

Lowering of the vdW-DF-cx energy barrier attributes to the nature of electronic interaction incorporated in the xc-functionals and the distinction of optimised structures. To understand these, we calculate ${\rm{\Delta }}E(\theta )$ of the vdW-DF-cx- structures for *θ* = 0° to 60°, due to a symmetry of the energy curves, with applications of the following xc-functionals: vdW-DF-cx, PBEsol, vdW-DF-cx-ex (non-local correlation is turned off), and PBEsol-ex (PBEsol density gradient correlation is turned off) (see Fig. 2). The electronic interaction contribution can be observed by comparing ${\rm{\Delta }}{E}^{{\rm{PBEsol}}({\rm{vdW}}-{\rm{Str}})}$ with $\,{\rm{\Delta }}{E}^{{\rm{vdW}}-{\rm{DF}}-{\rm{cx}}}$. At $\theta =\,60^\circ$, ${\rm{\Delta }}{E}^{{\rm{PBEsol}}({\rm{vdW}}-{\rm{Str}})}$ is $\sim 7.0$ meV higher than ${\rm{\Delta }}{E}^{{\rm{vdW}}-{\rm{DF}}-{\rm{cx}}}$ which reflects the fact that electronic non-local interaction lowers the rotational potential energy ${\rm{\Delta }}{E}^{{\rm{PBEsol}}-{\rm{ex}}({\rm{vdW}}-{\rm{Str}})}$ .Furthermore, since vdW-DF energy is split into semi-local (${E}_{xc}^{0}$) and truly non-local (${E}_{xc}^{nl}$) constituents, i.e. ${E}^{{\rm{vdw}}}={E}_{xc}^{0}+{E}_{xc}^{nl}$, and the semi-local part of the vdW-DF-cx is chosen such that its exchange component resemble that of GGA-type xc-functional in the small-to-medium scaled density gradient regime^32^. One can read a difference between semi-local correlation and non-local correlation effects by comparing ${\rm{\Delta }}{E}^{{\rm{PBEsol}}({\rm{vdW}}-{\rm{Str}})}-{\rm{\Delta }}{E}^{{\rm{PBEsol}}-{\rm{ex}}({\rm{vdW}}-{\rm{Str}})}$ with ${\rm{\Delta }}{E}^{{\rm{vdW}}-{\rm{DF}}-{\rm{cx}}}-{\rm{\Delta }}{E}^{{\rm{vdW}}-{\rm{DF}}-{\rm{cx}}-{\rm{ex}}}$. PBEsol correlation is positive at *θ* = 10°, 20° while vdW-DF-cx correlation is negative at all rotational angles. Moreover, at these beginning rotational angles, the exchange parts of both semi-local and non-local xc-functionals are almost the same. The non-local correlation becomes crucially important at the moderate rotational angles (*θ* = 40°, 50°, 60°). The structural contribution can be perceived by comparing ${\rm{\Delta }}{E}^{{\rm{PBEsol}}}$ with ${\rm{\Delta }}{E}^{{\rm{PBEsol}}({\rm{vdW}}-{\rm{Str}})}$. This contribution attributes to: *i*. unit cell deformation (Fig. 3(a,b)), *ii*. I-displacement (octahedron distortion) (Fig. 3(c–e)), *iii*. MA arrangement and its internal distortion, and *iv*. hydrogen bonding (Fig. 4(b)). For instance, at $\theta =\,60^\circ$, ${\rm{\Delta }}{E}^{{\rm{PBEsol}}}-{\rm{\Delta }}{E}^{{\rm{PBEsol}}({\rm{vdW}}-{\rm{Str}})}\cong 3.1\,{\rm{meV}}$ (Fig. 2) is a consequence of: *i*. short PBE-optimised lattice vectors comparing to those of vdW-DF-cx-optimised structure while a difference in unit cell shearing is negligible, *ii*. notably large PBE-optimised I_2_ atomic displacement in opposite of [100] direction comparing to that of vdW-DF-cx-optimised structures. Moreover, at this angle, only Hn_1_ – I_1_^(bc)^, Hn_2_ – I_1_^(c)^, and Hn_3_ – I_2_^(a)^ hydrogen bonds play the role (Fig. 4(b)), and those of PBEsol-optimised and vdW-DF-cx-optimised structures are almost the same. It is important to note here that the internal distortion of MA is negligible. Thus, ${\rm{\Delta }}{E}^{{\rm{PBEsol}}} > {\rm{\Delta }}{E}^{{\rm{PBEsol}}({\rm{vdW}}-{\rm{Str}})}$ mainly attributes to unit cell and octahedron deformations. The lowering of rotational potential barrier is predominated by the non-local interaction keeping H-atoms and I-atoms further apart from each other.

The broadening of vdW-DF-cx rotational potential energy curve can be related to an abrupt change of H-atom positions, illustrated in Fig. 4(a), accompanying with I_1_ and I_3_ displacements in [001] and [100] directions, respectively. These differences in optimised structures reflect the noticeable distinction in force field calculated by semi-local and non-local xc-functionals. This is emphasised by the totally difference in changes of C-N axis altitudinal angle when the MA undergo on-axis rotation shown in Fig. 3(f). This indicates the need for theoretical revision of dynamical properties of this material in which the truly non-local interaction is included as its importance has recently been realised in spin-polarised system^35^ and nonmagnetic transition metals^36^.

Furthermore, we examine the rotational potential energy when there are constraints imposed in structural relaxation process. The results are shown in Fig. 1(c). The vdW-DF-cx and PBEsol rotational energy barriers are 37.7 meV and 50.7 meV, respectively when the cell parameters are kept at the lowest energy configuration (B), and 50.6 meV and 47.0 meV, respectively, when both cell parameters and I-atomic positions are kept at the lowest energy configuration (C). We also calculate the free on-axis rotational frequencies corresponding to such energy barriers. By obeying the transition-state theory^30^, the frequency is given by:

$$\nu =\frac{{k}_{B}T}{h}{e}^{-{\rm{\Delta }}E/{k}_{B}T}$$

when Δ*E* is the rotational energy barrier. The obtained frequencies, at T = 330 K, for the relaxation model A, B, and C are 3.71 (2.60) THz, 1.83 (1.16) THz, and 1.16 (1.32) THz, respectively, for vdW-DF-cx (PBEsol) calculations. These rotational frequencies may help one experimentally justifies which MA rotational mechanism it is, if the local rotational frequency could be experimentally probed.

**Figure 5:**
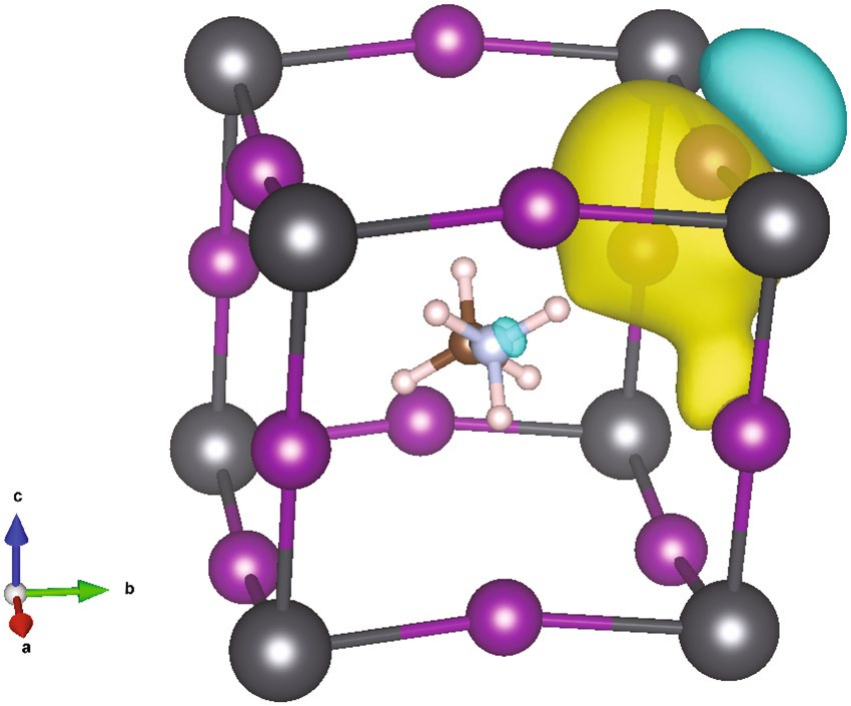
An isosurface of MLWF with the isosurface value = $\frac{\pm 1}{\sqrt{{\boldsymbol{V}}}}$ calculated with vdW-DF-cx xc-functional at *θ* = 60° where V is a unit cell volume in Å^3^. Yellow and turquoise represent positive and negative values, respectively.

As we have seen in Fig. 4(b), even in this high symmetry phase the complexity of hydrogen bonding takes place in this material. Especially when MA undergoes rotation, the hydrogen bonding associated with a specific H-atom can be turned on/off or switches to bond with the other I-atom depending on the rotational angle. For example, Hn_1_ strongly bonds with I_3_^(ab)^ at θ ∈ [0°, 40°] then switches to strongly bond with I_1_^(bc)^, (a criterion used for strong H-bond is H ··· I ≤ 3 *Å*). To illustrate that, we perform the maximally localised Wannier function (MLWF) analysis by projecting the 25 bands Bloch manifold onto 12 atom-centred MLWFs manifold^37^. The complete MLWFs calculated at *θ* = 0°, 40°, 60° are given in supplementary information. Here, it is worth highlighting a MLWF that localised in vicinity of the I_1_^(bc)^ obtained at *θ* = 60°. This MLWF mainly has I-p lone pair atomic orbital character and, also, shows covalent bonding character with the I_3_^(ab)^ assisted by Hn_1_, see Fig. 5. Note that bond character identification of the hydrogen bond supported I^−^ − I^−^ interaction is based on the basic idea of the quantum theory of atoms in molecules formulated by RFW. Bader^38^ that the I-p lone pair of the I_1_^(bc)^ becomes delocalized into vicinity of the I_3_^(ab)^ instead of as localized as those of the other I-atoms. This result is the first direct theoretical evidence supporting the recently experimental observation of interstitial iodide migration that leads to I_2_ formation in tetragonal MAPbI_3_ via $2{{\rm{I}}}^{-}\to \,{{\rm{I}}}_{2}+2{e}^{-}$ and then material degradation with I_2_ as a product^39,40^. Moreover, recent experiment has revealed that I_2_ can further accelerate the degradation of the iodide perovskites^41^. Notably, our results show that I – I covalent bonding is supported by hydrogen bond formed when the structure is at the top of rotational potential hill which mean that I_2_ formation is induced by MA rotation activated by thermal energy.

## Conclusions

We have performed density functional theory (DFT) based calculations to investigate the equilibrium atomic configurations of the pseudocubic MAPbI_3_ hybrid perovskite with respect to on-axis coherent methylammonium rotation. First, we assess the accuracy of a number of semi-local and non-local van der Waals exchange correlation density functionals (xc-functional) by calculating the structural parameters and compare them to the experimental values. According to the results in Table 1, vdW-DF-cx xc-functional shows the best overall performance for structural property DFT calculation of this material. Second, we report details of structural and energetic responses to the MA rotation obtained through the full structural relaxations. Comparison of results calculated with application of vdW-DF-cx with those of PBEsol xc-functionals shows noticeable lowering and broadening of vdW-DF-cx rotational potential energy curve. The non-local vdW correlation becomes crucial at the rotational angles (at θ = 40°, 50°, 60°) where the potential energy is much higher than the lowest energy (at *θ* = 0°). That manifest an inevitability of including truly non-local xc-functional in DFT study of this system. Also, the rotational potential barriers and corresponding rotational frequencies are calculated for the cases that the unit cell is fixed, and both unit cell and I-atom positions are fixed in the relaxation processes. Finally, a maximally localised Wannier function analysis reflects that the I – I covalent bonding can be formed at *θ* = 60° with the assistance of hydrogen bonding. This can lead to I_2_ formation and material degradation.

## Methods

Static DFT calculation is performed with the pseudopotential and plane wave method implemented in Quantum-Espresso package^42^. The ion cores are represented by original scalar relativistic ultrasoft pseudopotentials taken from PSlibrary project^43^. Spin-orbit coupling (SOC) effects are not included in this work, as previous study suggested that SOC does not significantly affect ground state properties of this material^44^. The kinetic energy cut-off is set to 70 Ry. The unshifted k-point mesh is gridded into 6 × 6 × 6 by Monkhorst-Packs scheme^45^. This configuration yields convergence of total energy within 2 meV. The nonlocal vdW exchange correlation functionals considered in this work are as follows: rvv10^46^, vdW-DF^47,48^, vdW- DF2^49^, vdW-DF-ob86^50^ and vdW-DF-cx^51^. The Perdew-Burke-Ernzerhof (PBE)^52^ and PBEsol^53^ functionals are also applied for comparison. The relaxation algorithm of atoms and lattice parameters used is Broyden-Fletcher-Goldfarb-Shanno (BFGS) algorithm with a force/atom tolerance equal to 0.001 Hartree/Bohr. The initial structure is the lowest enthalpy configuration reported by Brivio *et al*. study^14^ (available online^54^) re-optimised with our settings. The calculations of exchange energies are achieved by setting of the Quantum-Espresso input variable, i.e. input_dft, as following: input_dft = ‘sla + pw + cx13’, and input_dft = ‘sla + pw + psx + nogc’ for vdW-DF-cx and PBEsol exchange (with local correlation) density functionals respectively. For maximally localised Wannier function (MLWF) calculation, the k-space is meshed by 8 × 8 × 8 grid. Twenty-five Bloch wave functions are projected onto twelve atom-centred MLWFs with convergent criteria of the spread function equal to 10^−10^*Å*^2^.

## Electronic supplementary material

Supplementary
