# Supplementary material for: The crucial role of density functional nonlocality and on-axis CH3NH3 rotation induced I2 formation in hybrid organic-inorganic CH3NH3PbI3 cubic perovskite
Source: Sci Rep. 2018 Sep 3;8:13161. doi: 10.1038/s41598-018-31462-x (PMC6120889; doi:10.1038/s41598-018-31462-x)
Supplement: Supplementary file 1 — Supplementary [file 41598_2018_31462_MOESM1_ESM.pdf]

Supplementary information

The crucial role of density functional nonlocality and on-axis CH_3NH_3 rotation induced I_2 formation in hybrid organic-inorganic $CH_3NH_3PbI_3$ cubic perovskite

Rakchat Klinkla[1,2], **Vichawan Sakulsupich**[1,2], **Teerachote Pakornchote**[1,2], **Udomsilp Pinsook**[1,2], **and Thiti Bovornratanaraks**[1,2*]

[1]Extreme Conditions Physics Research Laboratory, Physics of Energy Materials Research Unit, Department of Physics, Faculty of Science, Chulalongkorn University, Bangkok 10330, Thailand
[2]Thailand Center of Excellence in Physics, Commission on Higher Education, 328 SiAyutthaya Road, Bangkok 10400, Thailand.
[*]Thiti.b@chula.ac.th

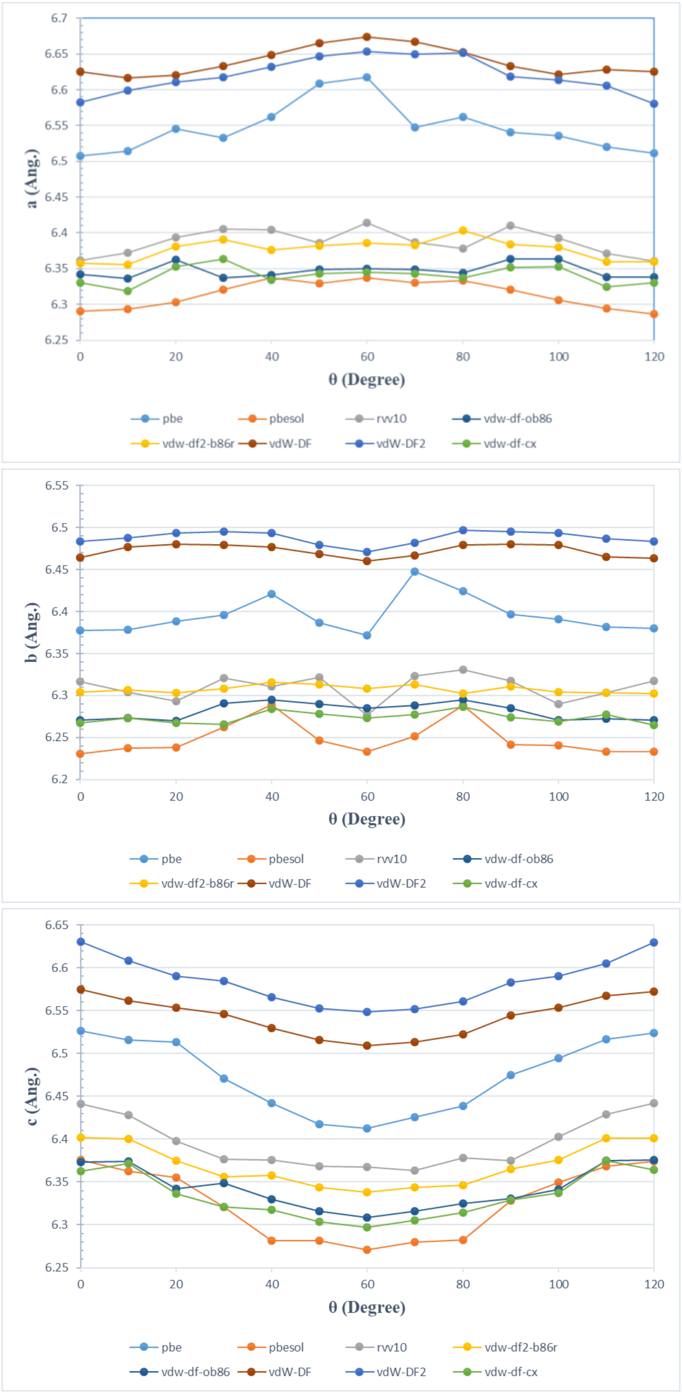

Figure S. 1 Magnitudes of three optimized primitive vectors obtained by performing full structural relaxation, which is there is no any constraint, with applications of a number of semi-local and non-local exchange correlation functionals as a function of rotation angle. The results show inconsistency of PBE, vdW-DF, vdW-DF2 exchange correlation functionals with the others.

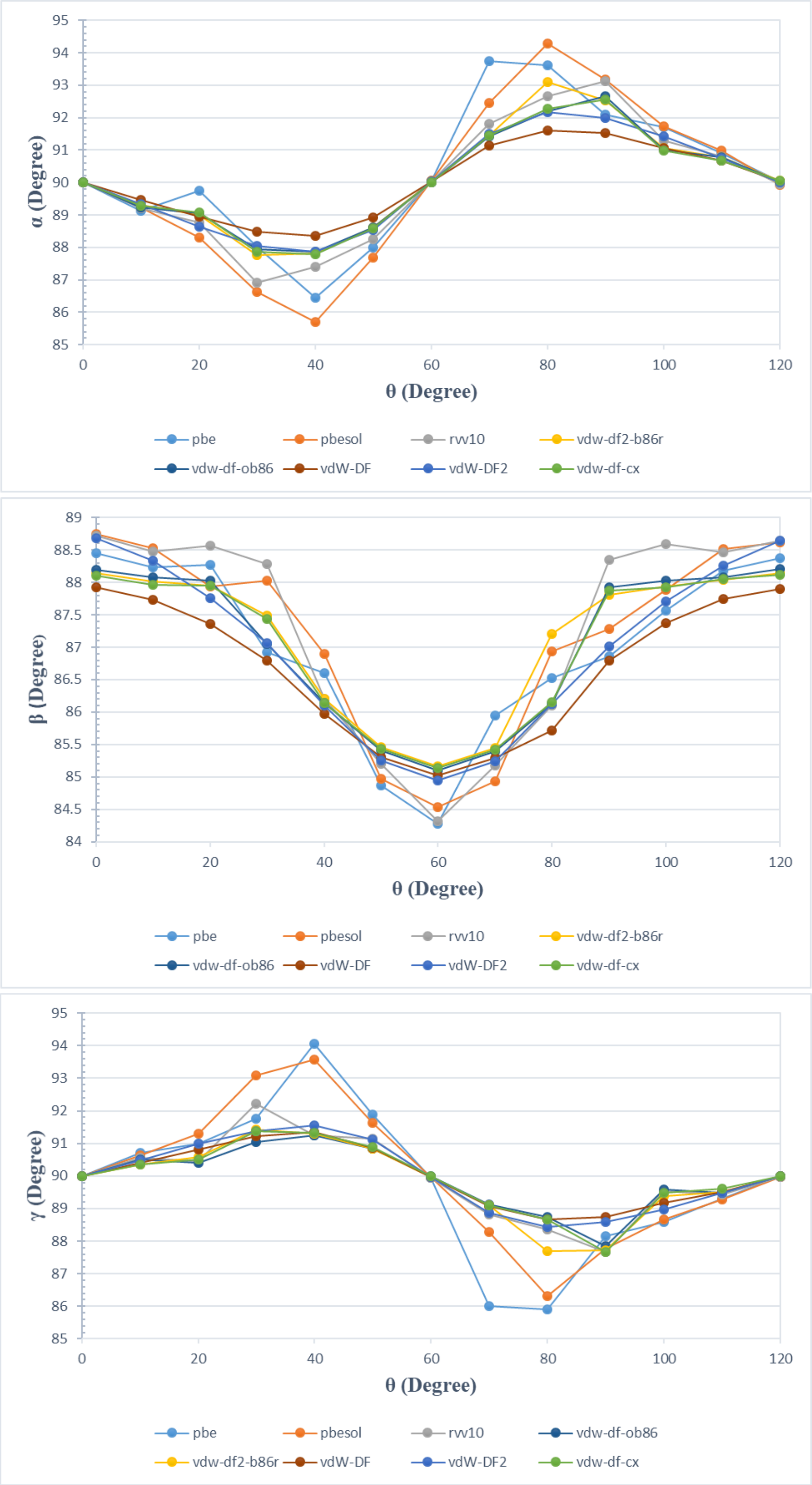

Figure S. 2 Magnitudes of three angles between the primitive vectors obtained by performing full structural relaxation, which is there is no any constraint, with applications of a number of semi-local and non-local exchange correlation functionals as a function of rotation angle.

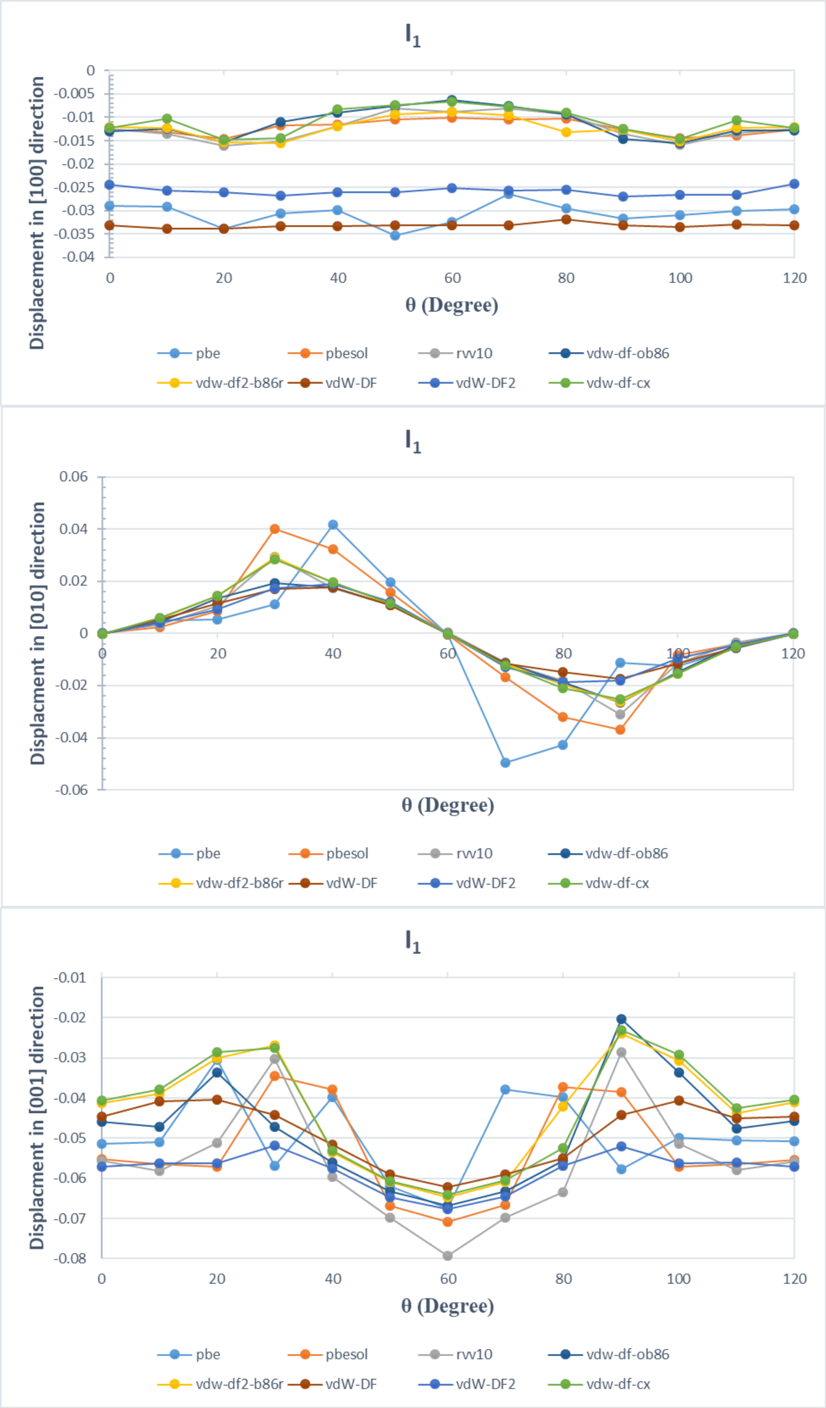

Figure S. 3 Displacements of I_1-atom from the mid-point of a primitive vector "**a**" in three crystallographic directions obtained by performing full structural relaxation, which is there is no any constraint, with applications of a number of semi-local and non-local exchange correlation functionals as a function of rotation angle.

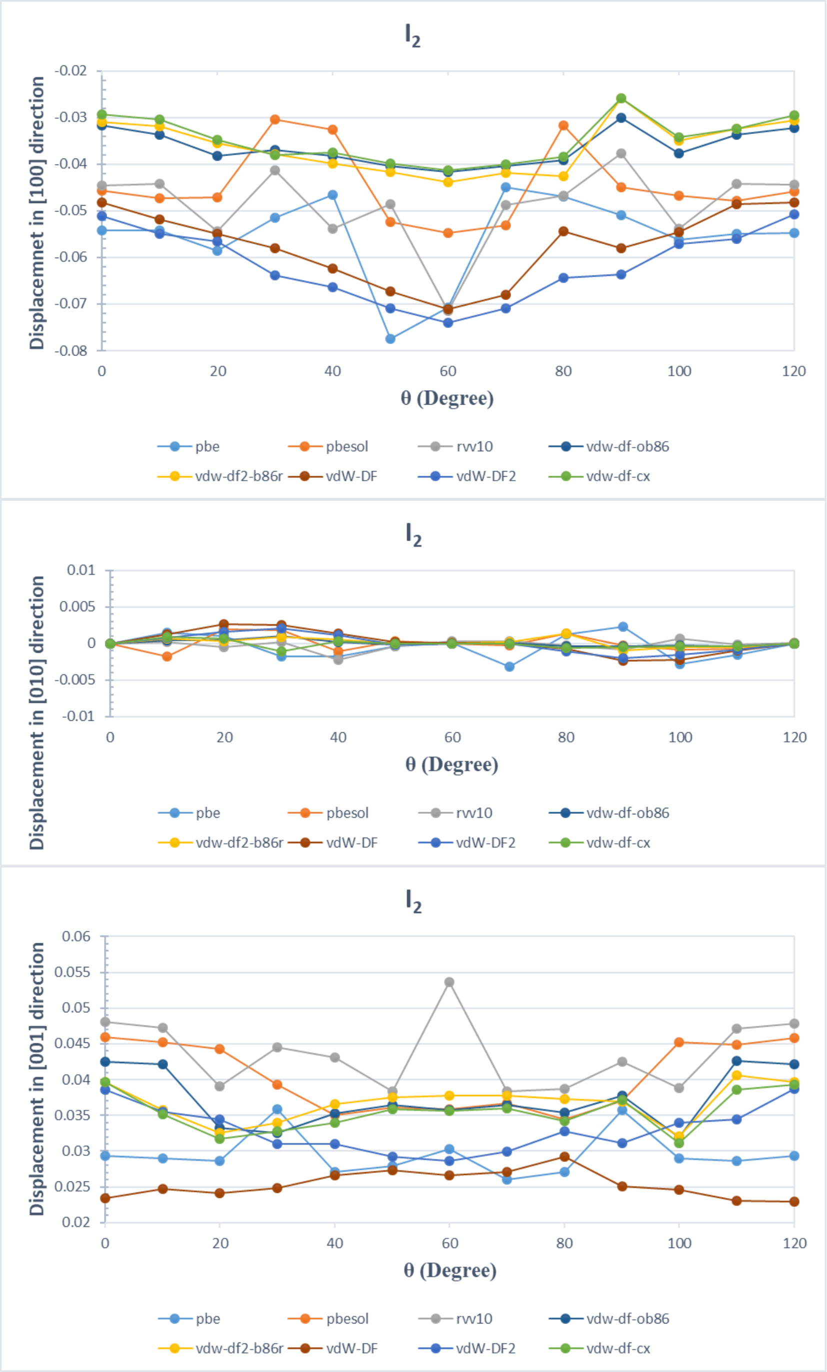

Figure S. 4 Displacements of I_2-atom from the mid-point of a primitive vector “**b**” in three crystallographic directions obtained by performing full structural relaxation, which is there is no any constraint, with applications of a number of semi-local and non-local exchange correlation functionals as a function of rotation angle.

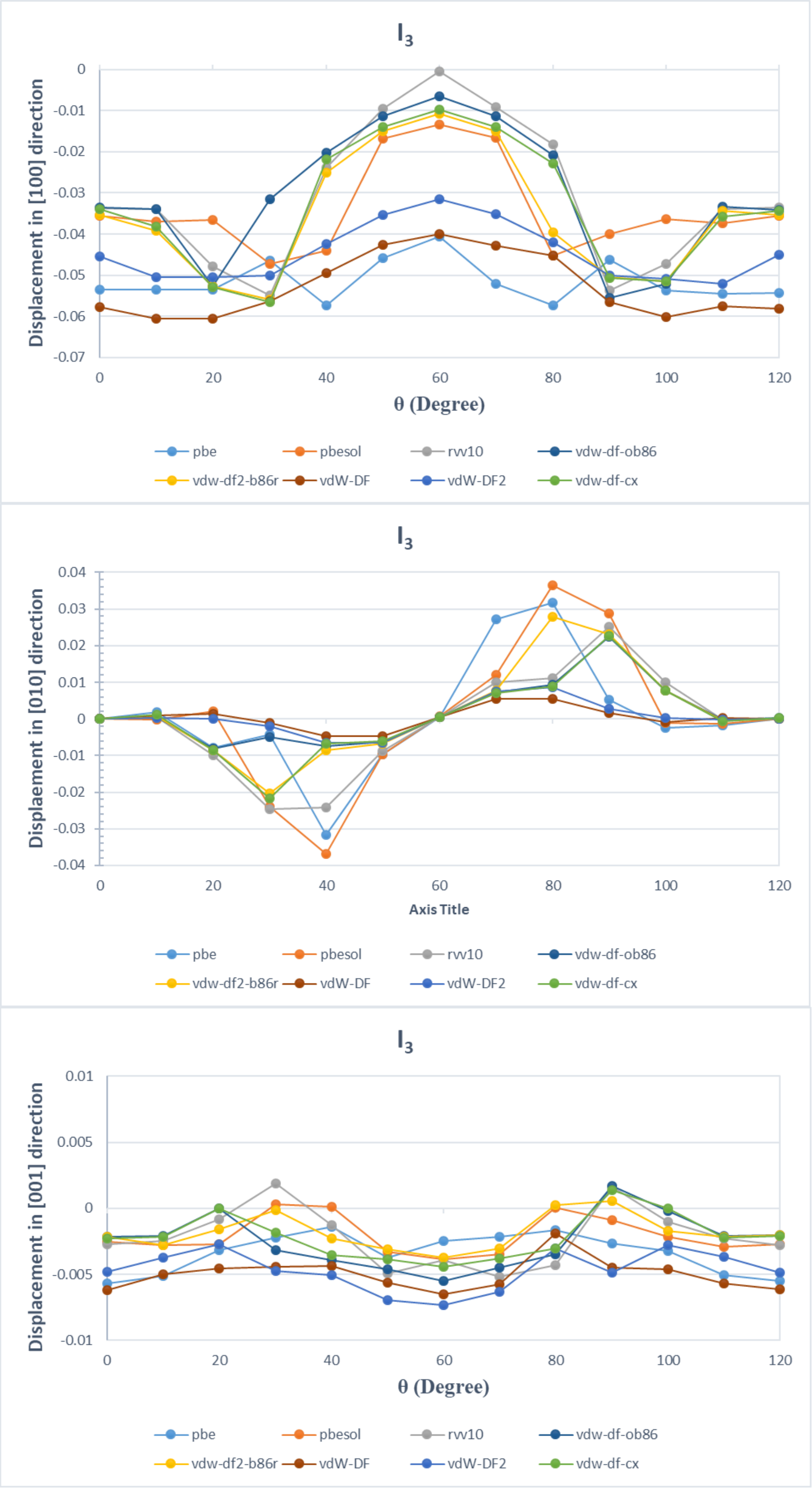

Figure S. 5 Displacements of I3-atom from the mid-point of a primitive vector "**c**" in three crystallographic directions obtained by performing full structural relaxation, which is there is no any constraint, with applications of a number of semi-local and non-local exchange correlation functionals as a function of rotation angle.

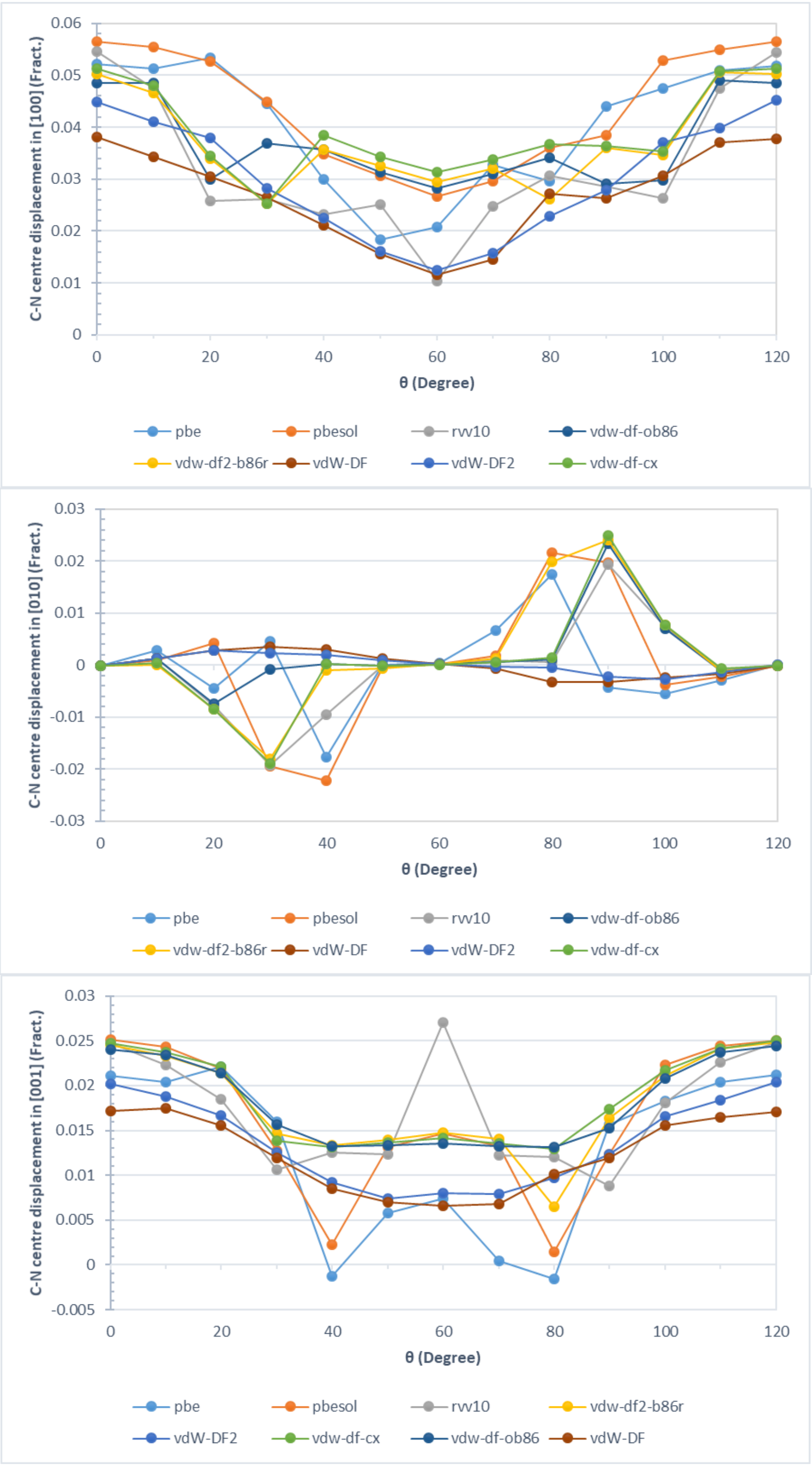

Figure S. 6 Deviation of C-N centre from [0.5, 0.5, 0.5] in three crystallographic directions obtained through full structural relaxations.

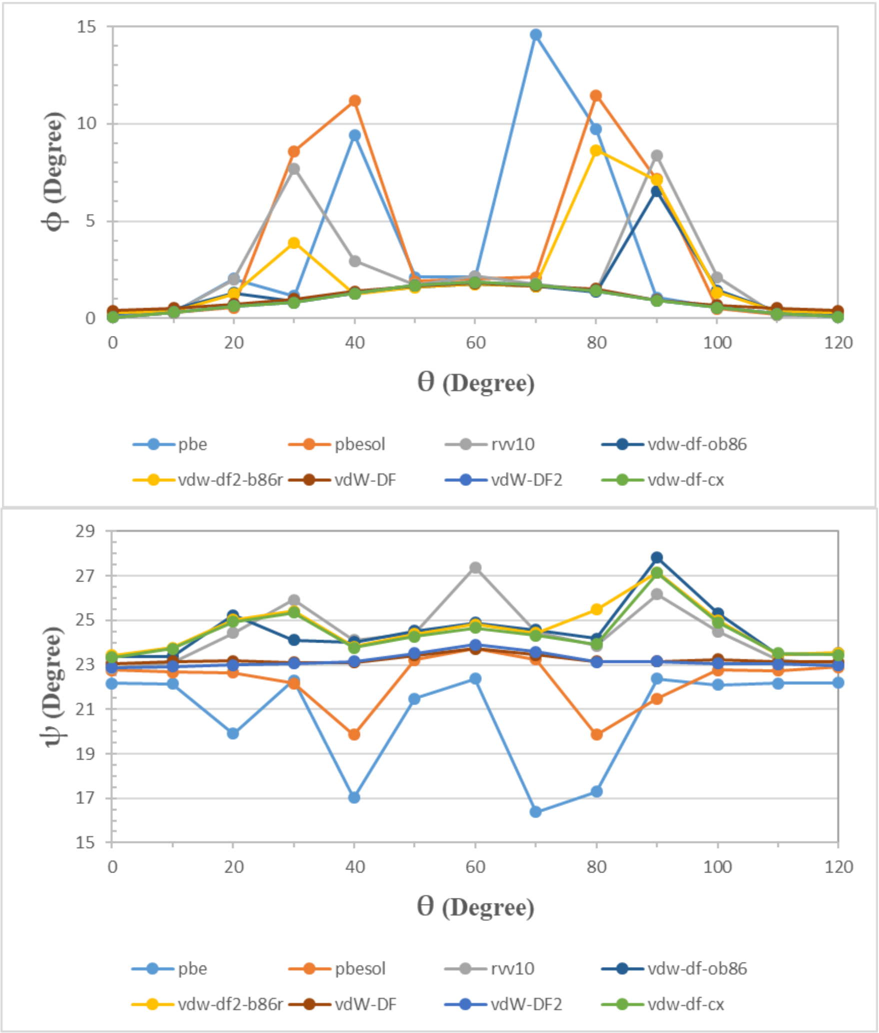

Figure S. 7 Arrangement of C-N axis obtained through full structural relaxations. Measures of ϕ and ψ are corresponding to Fig. 1(b) in the main text.

PBEsol: $\theta = 0°$

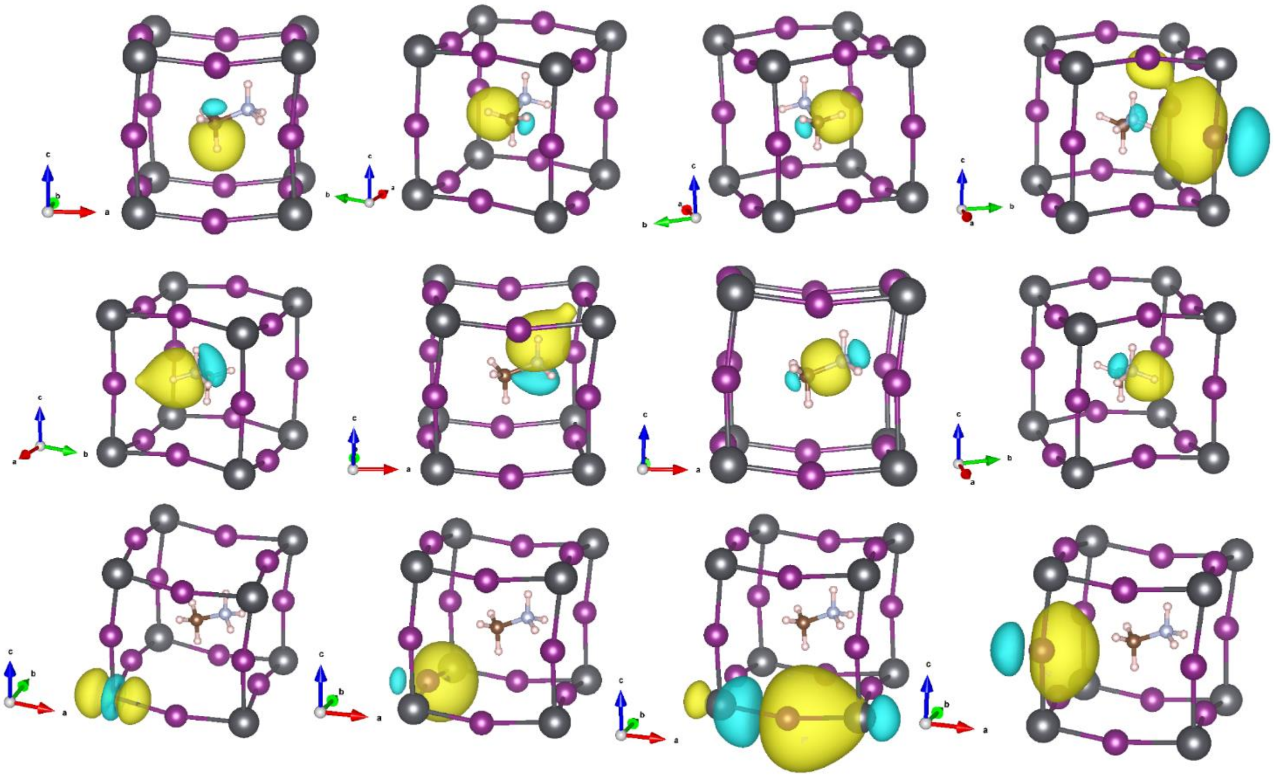

PBEsol: $\theta = 40°$

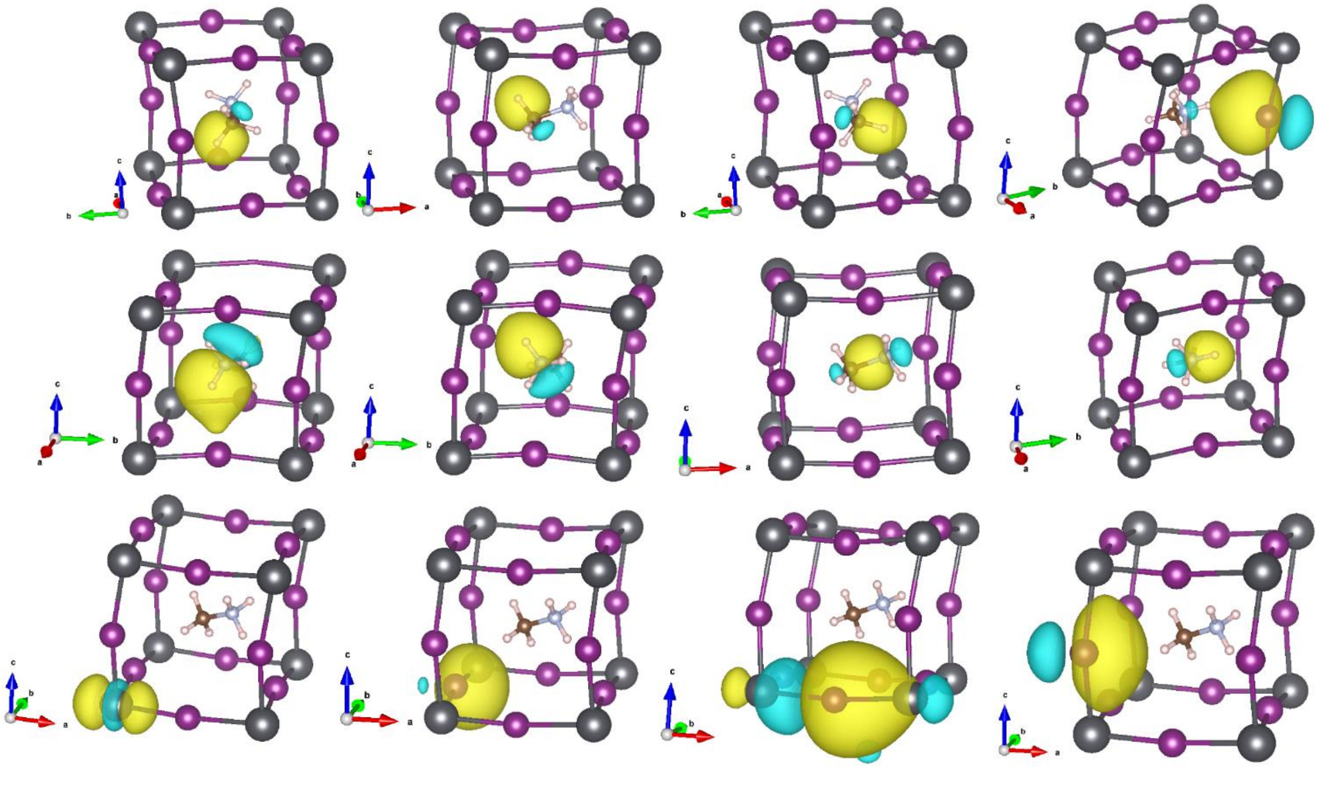

PBEsol: $\theta = 60^{\circ}$

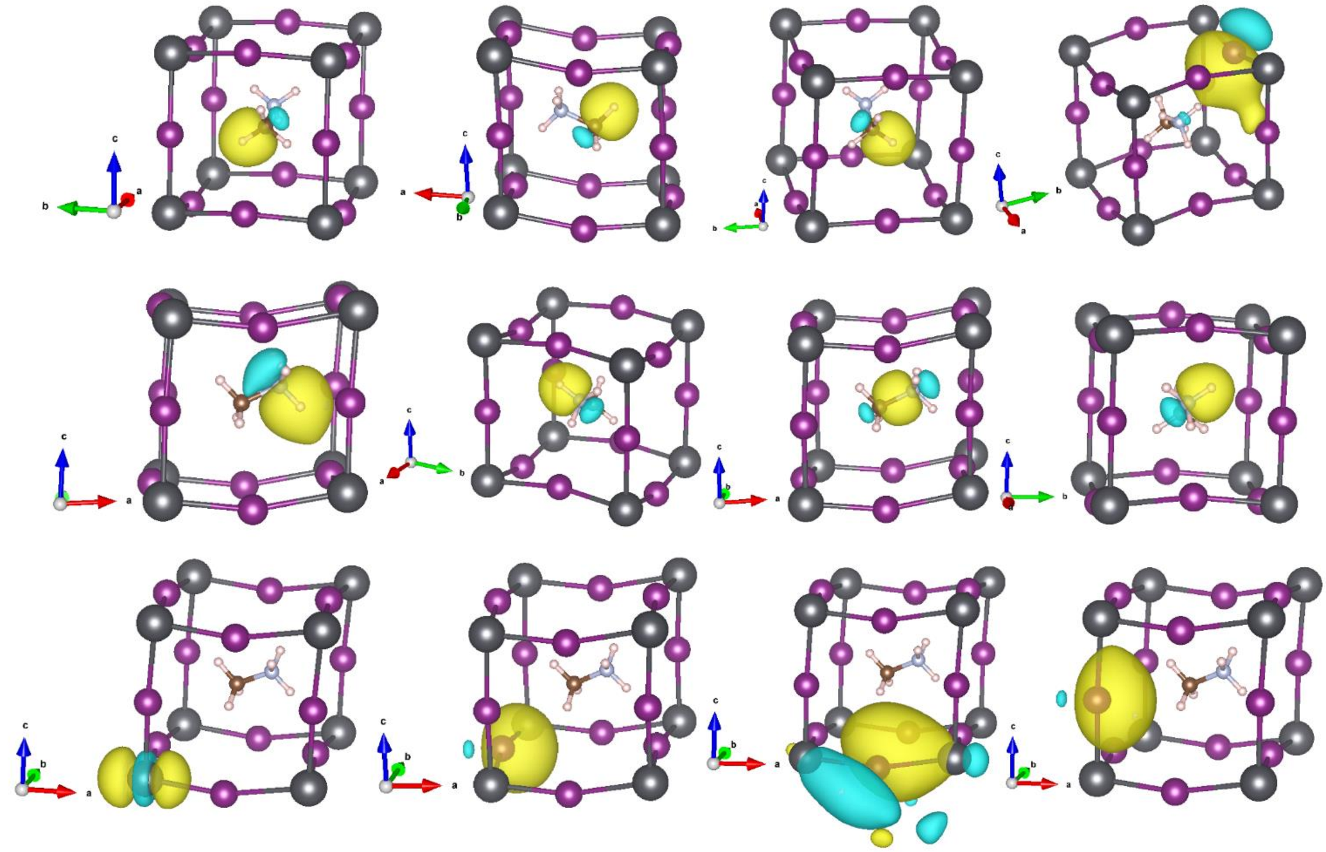

vdW-DF-cx: $\theta = 0^{\circ}$

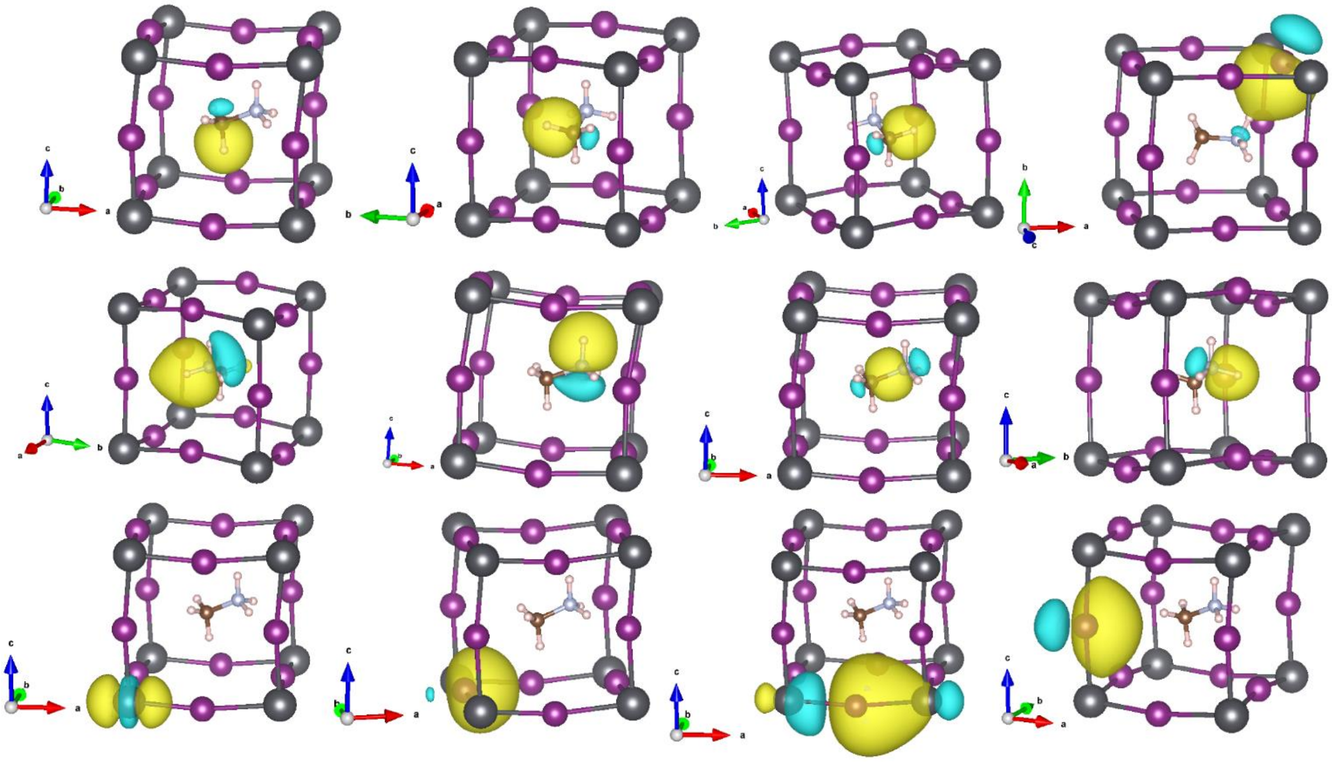

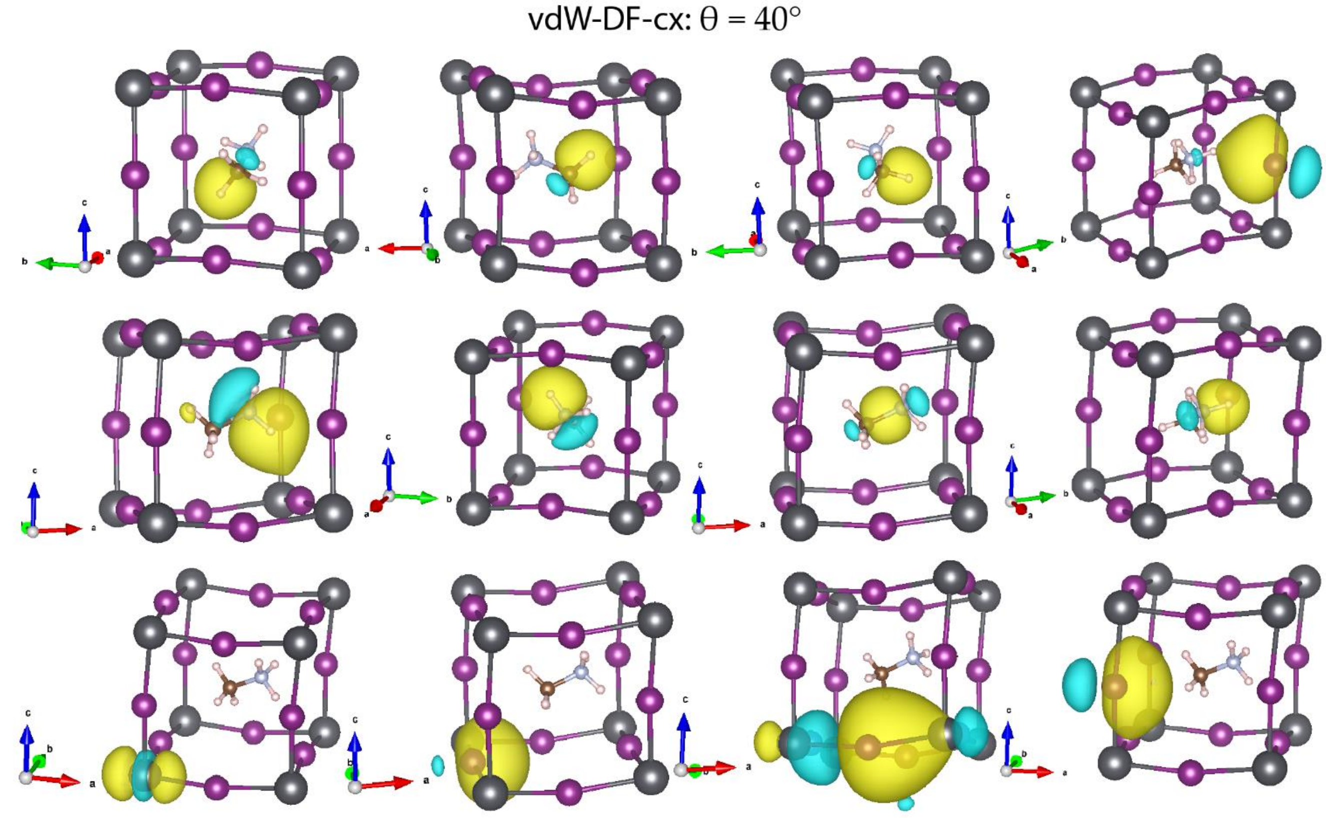

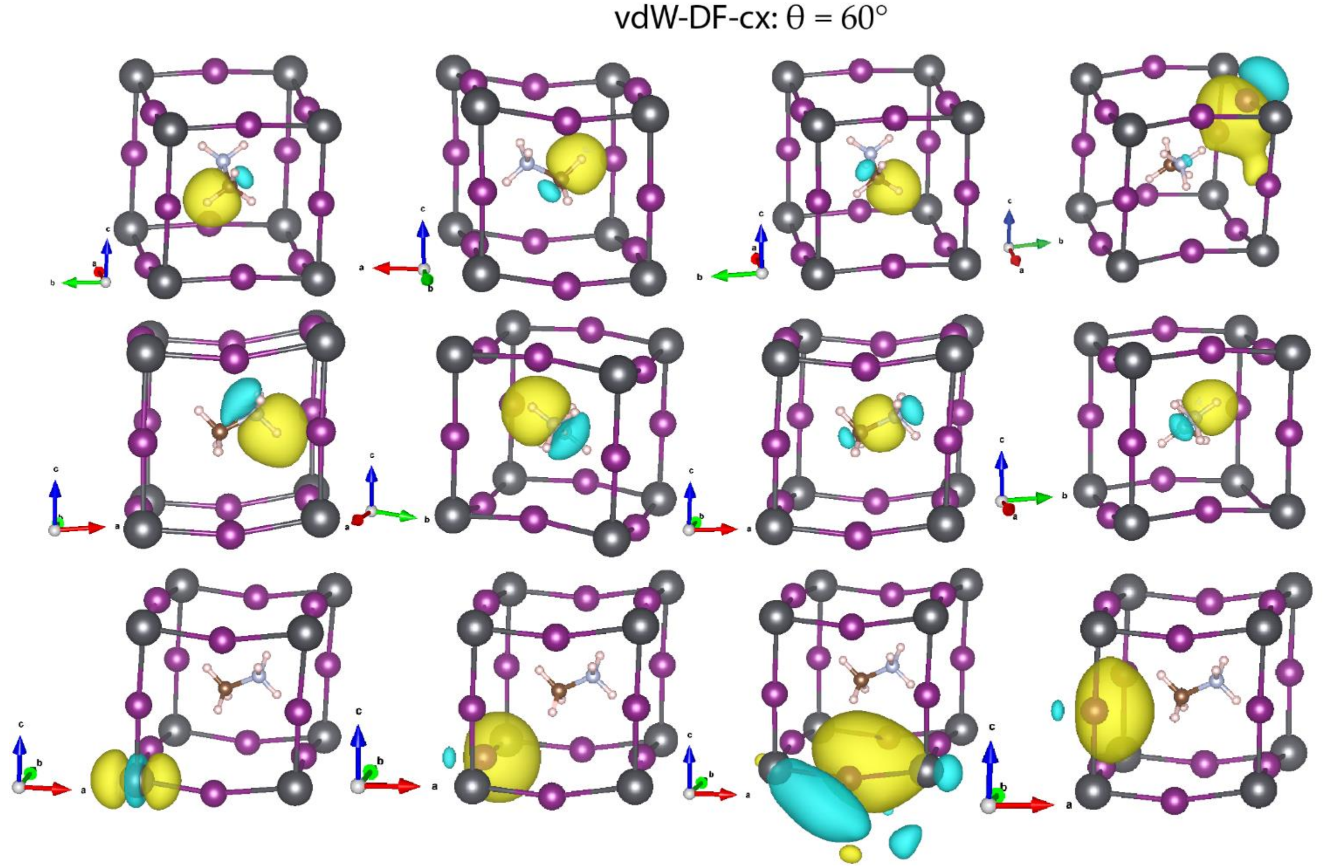

Figure S. 8 Complete isosurfaces of maximally localised Wannier functions of the optimized structures calculated with applications of PBEsol and vdW-DF-cx exchange correlation functionals at $\theta = 0^{\circ}, 40^{\circ}, 60^{\circ}$.
